# Accuracy and Precision of Energy Expenditure, Heart Rate, and Steps Measured by Combined-Sensing Fitbits Against Reference Measures: Systematic Review and Meta-analysis

**DOI:** 10.2196/35626

**Published:** 2022-04-13

**Authors:** Guillaume Chevance, Natalie M Golaszewski, Elizabeth Tipton, Eric B Hekler, Matthew Buman, Gregory J Welk, Kevin Patrick, Job G Godino

**Affiliations:** 1 Barcelona Institute for Global Health ISGlobal Barcelona Spain; 2 Herbert Wertheim School of Public Health and Longevity Science University of California, San Diego La Jolla, CA United States; 3 Center for Wireless & Population Health Systems University of California, San Diego La Jolla, CA United States; 4 Department of Statistics Northwestern University Evanston, IL United States; 5 Exercise and Physical Activity Resource Center University of California, San Diego La Jolla, CA United States; 6 School of Nutrition & Health Promotion Arizona State University Phoenix, AZ United States; 7 Department of Kinesiology Iowa State University Ames, IA United States; 8 Laura Rodriguez Research Institute Family Health Centers of San Diego San Diego, CA United States

**Keywords:** wearables, activity monitors, physical activity, validity, accelerometry

## Abstract

**Background:**

Although it is widely recognized that physical activity is an important determinant of health, assessing this complex behavior is a considerable challenge.

**Objective:**

The purpose of this systematic review and meta-analysis is to examine, quantify, and report the current state of evidence for the validity of energy expenditure, heart rate, and steps measured by recent combined-sensing Fitbits.

**Methods:**

We conducted a systematic review and Bland-Altman meta-analysis of validation studies of combined-sensing Fitbits against reference measures of energy expenditure, heart rate, and steps.

**Results:**

A total of 52 studies were included in the systematic review. Among the 52 studies, 41 (79%) were included in the meta-analysis, representing 203 individual comparisons between Fitbit devices and a criterion measure (ie, n=117, 57.6% for heart rate; n=49, 24.1% for energy expenditure; and n=37, 18.2% for steps). Overall, most authors of the included studies concluded that recent Fitbit models underestimate heart rate, energy expenditure, and steps compared with criterion measures. These independent conclusions aligned with the results of the pooled meta-analyses showing an average underestimation of −2.99 beats per minute (*k* comparison=74), −2.77 kcal per minute (*k* comparison=29), and −3.11 steps per minute (*k* comparison=19), respectively, of the Fitbit compared with the criterion measure (results obtained after removing the high risk of bias studies; population limit of agreements for heart rate, energy expenditure, and steps: −23.99 to 18.01, −12.75 to 7.41, and −13.07 to 6.86, respectively).

**Conclusions:**

Fitbit devices are likely to underestimate heart rate, energy expenditure, and steps. The estimation of these measurements varied by the quality of the study, age of the participants, type of activities, and the model of Fitbit. The qualitative conclusions of most studies aligned with the results of the meta-analysis. Although the expected level of accuracy might vary from one context to another, this underestimation can be acceptable, on average, for steps and heart rate. However, the measurement of energy expenditure may be inaccurate for some research purposes.

## Introduction

### Background

Although it is widely recognized that physical activity is an important determinant of health [1,2], assessing this complex behavior is a considerable challenge [3-5]. Tools for objective assessment of the frequency, intensity, and duration of physical activity in adults and children have largely been developed for short-term use within research or public health surveillance environments [6,7]. However, recent advances in microtechnology, data processing, wireless communication, and battery capacity have resulted in the proliferation of low-cost, noninvasive, wrist-worn devices with attractive designs that can be easily used by consumers to track their physical activity over long periods [8].

The latest generation of consumer-level activity monitors is typically multi-sensor devices that use *triaxial accelerometry* to measure movement and *photoplethysmography* to measure heart rate (ie, number of beats per minute [bpm]). Importantly, a combined-sensing approach to measuring physical activity may address many of the limitations of using either accelerometry or photoplethysmography alone [9,10]. The combination of these data streams through branched equation modeling or machine learned algorithms might result in a more accurate assessment of physical activity [11,12].

The expanding use of consumer-level activity monitors in population and clinical health research has led to an array of independent studies aimed at evaluating the validity of various metrics. No devices have received more attention than those manufactured by Fitbit (Fitbit Inc). From community-based health interventions that aim to motivate individuals to increase their physical activity level to interventions that aim to improve patient–health professional interactions, Fitbits are likely the most widely used [13,14]. Hence, a major concern for consumers and researchers alike is understanding the extent to which Fitbits provide accurate estimates of physical activity.

Several studies have evaluated the validity of different versions of Fitbits in estimating energy expenditure, intensity, heart rate, or steps, mostly in controlled laboratory settings [15] and a limited amount in free-living conditions [16]. Moreover, there have been 4 systematic reviews have been conducted to examine the accuracy of measures derived from consumer-level activity monitors in general [17-19] and from Fitbits specifically [20]. Taken together, these reviews conclude that Fitbit devices accurately measure steps and heart rate, whereas estimates of energy expenditure are less than optimal and tend to be underestimated. These reviews also spotted large variations around the estimates, highlighting potential sources of undetermined heterogeneity.

Although previous systematic reviews have been informative, several limitations exist within these reviews. First, 3 of the 4 systematic reviews [18-20] have compared Fitbits with questionable criterion measures, such as other wearable devices (ie, accelerometers), instead of ground truth or reference measures of energy expenditure [21], heart rate [22], or steps [23]. Second, all previous reviews have included older versions of the Fitbit that do not use photoplethysmography combined with accelerometry, which are (1) less likely to be used in future studies and (2) likely to result in more bias than the more recent Fitbits [11]. Third, there is yet to be a quantitative synthesis of the validity of recent Fitbits through a meta-analysis. Such meta-analytical work could notably help identify sources of heterogeneity in the validity of these devices for different outcomes and contexts of use.

### Objective

The purpose of this systematic review and meta-analysis is to examine, quantify, and report on the current state of evidence for the analytical validity of energy expenditure, heart rate, and steps measured by recent combined-sensing Fitbits. On the basis of the existing literature, we expected some form of accuracy for the estimation of steps and heart rate and a lack of precision for energy expenditure. No hypotheses were formulated for the quantitative part of this study (ie, meta-analysis).

## Methods

The protocol was registered with PROSPERO (International Prospective Register of Systematic Reviews; CRD42020161937) and is reported according to the PRISMA-P (Preferred Reporting Items for Systematic Review and Meta-Analyses Protocols) [24] guidelines (Multimedia Appendix 1). All study materials, including not only code and data but also the supplemental materials, are available on the *Open Science Framework* [25].

### Search Strategy

A systematic review of the literature was conducted in 3 iterations to retrieve both published and unpublished studies [26]. The search was conducted using the PubMed and Embase databases from January 2015 (ie, commercialization of the first Fitbit device that included a heart rate monitor) to July 2021. The gray literature was also inspected through Open Grey (Multimedia Appendix 2). In the second iteration, studies were also sourced from previously published systematic reviews [17-20]. In the third iteration, reference lists within the studies included in the previous iterations were examined. Published conference abstracts were also included if sufficient detail was reported to assess study quality. In cases where information was missing, attempts were made to contact the authors. Study selection was performed by one coder (GC) and checked by an independent second coder (NMG). Any discrepancies were identified and resolved. No language restrictions were applied.

### Criteria for Study Inclusion

Studies that simultaneously reported outcome data from a Fitbit device (energy expenditure, heart rate, or steps) and a valid criterion measure were considered. Only studies that evaluated Fitbit devices that include a heart rate monitor (ie, *Charge HR 2015, Surge 2015, Blaze 2016, Charge 2 2016, Alta HR 2017, Ionic 2017, Versa 2018, Charge 3 2018, Inspire HR 2019, Versa 2 2019, Versa Lite Edition 2019, Charge 4 2020, Versa 3 2020, Sense 2020*, and *Inspire 2 2020*) were included. Valid criterion measures of energy expenditure included doubly labeled water or direct and indirect calorimetry; for heart rate, they included electrocardiograms, pulse oximeters, and specific chest-worn systems (eg, Polar), and for steps, direct observation was the only criterion (video recorded or not).

### Data Extraction and Management

Information about the study characteristics (authors, year of publication, design, sample size, and number of observations for each outcome), population characteristics (age, health conditions, and BMI), descriptive statistics, type of Fitbit, and features of the criterion measures were extracted. Finally, given (1) the heterogeneity of the protocols to test the validity of the Fitbit, (2) the multiple statistical strategies used to perform the analyses (eg, Bland-Altman analyses vs analysis of variance), and (3) the lack of consensus in the interpretation of these statistical outcomes (ie, to infer whether a device is valid), we also decided to retrieve the explicit conclusion of the authors when judging the particular validity or a device.

For the meta-analysis, the effect sizes extracted were the mean bias (ie, accuracy) and variance or SD (ie, precision) in kilocalories per minute (kcal per minute), bpm, and difference of steps per minute (steps per minute) between the Fitbit and criterion measures of energy expenditure, heart rate, and steps, respectively. It is important to note that kcal and steps are not always reported as a function of time (ie, per minute). Some authors prefer the total amount of kcal or steps recorded during a specific task or an entire protocol. To make the comparisons between studies and interpretation of the results possible, we retrieved the time spent during each protocol task. We then converted the absolute number of kcal and steps to kcal and steps per minute by dividing the mean bias and SD reported by the duration of each specific task in minutes. For example, a mean bias of 20 (SD 10) kcal recorded over a 3-minute task was converted to 6 (SD 3) kcal per minute.

These outcomes were extracted directly from eligible studies when available or computed using other reported statistics (ie, means, SDs, and correlations). If needed, the authors were contacted and asked to provide the necessary information. Data were extracted and coded by one coder (GC) and checked by a second coder (NMG). Discrepancies were identified and resolved by rereferencing the articles and reaching a consensus with a third author (JGG).

### Data Synthesis and Analyses

A specific meta-analytic framework was used for the analyses of agreement between the measures [27]. The main outcome of the Bland-Altman meta-analysis was the population limits of agreement between Fitbit devices and criterion measures of energy expenditure, heart rate, and steps. The population limits of agreement combine the bias of a test (ie, the average difference between the tested measure and a criterion measure) and the SD of these differences. The results from the individual studies were first converted into a standard format to conduct the meta-analysis, with bias captured as *Fitbit–criterion measure*. Outcomes were expressed in kcal per minute, bpm, and steps per minute for energy expenditure, heart rate, and steps, respectively.

The population limits of agreement were then computed to account for two sources of variation: the average within-study variation and the between-study variation. The computed population limits of agreement were typically wider (ie, more *conservative*) than those reported in other meta-analyses of Bland-Altman studies (for further explanations, refer to the study by Tipton and Shuster [27]). In this study, the pooled limits of agreement were calculated using δ±2√(σ^2^+τ^2^), where δ is the average bias across studies, σ^2^ is the average within-study variation in differences, and τ is the SD of bias across studies (a larger τ indicates higher variations in bias between studies). Both δ and σ^2^ were estimated using a weighted least squares model (similar to a random effects approach), and their SEs were estimated using robust variance estimation (RVE). RVE was used instead of model-based SEs as most of the studies included in our review used repeated measures designs without accounting for the correlation between measurements (ie, multilevel approach). The method of moments estimator was used for the τ parameter [28]. Measures of uncertainty were also included when interpreting the limits of agreement estimates by calculating the outer 95% CIs for pooled limits of agreement and adjusted repeated measurements, which were not properly adjusted for in individual studies [27]. Multiple effect sizes from the same study were also handled using the RVE method [29,30].

### Planned Sensitivity and Subgroup Analyses

Subgroup meta-analyses were performed for the following variables: (1) characteristics of the participants, including the presence of health conditions and age (<65 years and >65 years); (2) type of Fitbit device; (3) type of activity (eg, resting and sedentary activities, ambulation, and cycling); (4) intensity (ie, differences in light and moderate to vigorous intensity activities); and (5) study quality (ie, see the following sections). The limits between light- and moderate-intensity physical activity for the intensity variable were defined according to the Compendium of Physical Activities. For example, walking >3 mph or 5 km/h and cycling >7 mph or 11 km/h, or 150 W, were considered moderate to vigorous physical activity. A complete description and justification of these analyses are provided in the registered protocol.

### Quality Assessment (Risk of Bias)

A custom tool, developed based on a previous study using the COSMIN (Consensus-Based Standards for the Selection of Health Measurement Instruments) criteria [31], was used to assess study quality, including (1) sample size calculation justifying a reasonably large sample (N>50=1 point [32]), (2) peer reviewing (study peer reviewed=1 point), (3) appropriate placement of the device (device up to 3 finger widths above the wrist bone=1 point [33]), and (4) validation of only 1 device on the wrist (1 device at a time=1 point), thus providing a quality score between 1 (low) and 4 (high). Sensitivity analyses were performed for the primary meta-analyses (ie, average energy expenditure, heart rate, and steps) based on the risk of bias by removing the *high risk of bias* studies (quality score ≤1) from the analyses and outliers. Subgroup analyses were also conducted according to the potential moderators identified previously and when at least four comparisons between the Fitbits and criterion measures were available.

All analyses were conducted using the R statistical program (version 4.1.2; R Foundation for Statistical Computing). The R code (adapted from the study by Tipton and Shuster [27]) and all the data used in the meta-analyses are available on the web [25].

## Results

### Systematic Review

A total of 52 studies were included in the systematic review (see Multimedia Appendix 3 for the study flowchart). Among the 52 studies, 41 (79%) were included in the meta-analyses, representing 203 individual comparisons between Fitbit devices and a criterion measure (ie, n=117, 57.6% for heart rate, n=49, 24.1% for energy expenditure, and n=37, 18.2% for steps; see study flowchart in Multimedia Appendix 3). The participants (n=1628) were mostly young (only 8/52, 15% of studies included participants aged >65 years), without chronic diseases (47/52, 90% of studies), and with a mean BMI of 24.9 kg/m^2^ (range 21-34). Approximately 15% (8/52) of studies included participants with chronic conditions (ie, cardiac, respiratory, and Parkinson diseases and chronic pain). The included studies mostly tested the validity of the devices as part of formal and structured laboratory protocols (45/52, 87%; see the column *Protocol* in Table 1) instead of activities measured in free-living conditions.

Of the 52 studies, the Fitbit *Charge HR* was included in 27 (52%) studies, the *Surge* in 11 (21%) studies, the *Charge 2* in 10 (19%) studies, the *Blaze* and *Versa* in 3 (6%) studies each, and the *Ionic* and *Charge 3* in 1 (2%) study each. Of the 52 studies, Fitbits were compared with a criterion measure for heart rate in 32 (62%) studies, energy expenditure in 19 (37%) studies, and steps in 15 (29%) studies. According to our inclusion criteria, heart rate was mainly estimated using electrocardiograms (18/32, 56%) or Polar heart rate straps (14/32, 44%). Energy expenditure was estimated using indirect calorimetry in all studies except one, which used doubly labeled water. Steps were measured with video records for 57% (8/14) of studies and a manual hand counter for 43% (6/14) of studies.

Regarding the authors’ study conclusions, 63% (20/32), 79% (15/19), and 27% (4/15) of studies concluded that the estimations provided by the Fitbit devices were not optimally valid compared with the reference standards for heart rate, energy expenditure, and steps, respectively. Most studies (18/32, 56%) explicitly reported an underestimation of the Fitbits compared with criterion measures for heart rate in their conclusion (only one of the studies explicitly reported an overestimation of heart rate; the remaining studies did not explicitly provide a conclusion about under- or overestimation). Similarly, a large number of studies (6/15, 40%) reported an underestimation of the Fitbits compared with criterion measures for steps (only one of the studies explicitly reported an overestimation of steps; the remaining studies did not explicitly provide a qualitative conclusion about under- or overestimation). Results were mixed for energy expenditure, with 12% (6/52) of studies explicitly reporting an underestimation of this outcome for the Fitbit, and 10% (5/52) reporting an overestimation (one of the studies indicated mixed findings related to the intensity and the remaining did not explicitly provide a conclusion about under- or overestimation). See Table 1 for a detailed description of each study included in the systematic review.

**Table 1:** Outcomes of the systematic review (N=52).

Study	Participants	Fitbit	Outcomes	Criterion measures	Protocol	Statistics	Authors’ conclusion
Al-Kaisey et al [34]	Patients with cardiac conditions (N=12; observations=53,288)	Charge HR	HR^a^	ECG^b^ (DigiTrakXT)	24-hour monitoring within a cardiology department (usual routine)	Correlations; multilevel Bland-Altman analyses	Underestimation, particularly pronounced at HR ranges >100 bpm^c^; accuracy judged as insufficient
Baek et al [35]	Healthy adults; mean age 24 years (N=15)	Charge 2	HR	ECG (Philips StressVue)	Two 20-minute walking sessions on a treadmill (1 conventional walking and 1 Nordic walking)	Bland-Altman analyses; Lin concordance correlation coefficients; mean relative difference; paired *t* test	Accuracy judged as adequate for conventional walking and inadequate during Nordic walking
Bai et al [36]	Healthy adults; aged 19 to 60 years (N=39)	Charge HR	HR; EE^d^; steps not used in the MA^e^ (criterion measure=pedometer)	Polar heart rate chest strap; indirect calorimetry (Oxycon Mobile 5.0)	80-minute structured activity protocol (treadmill and free-living activities)	Bland-Altman analyses; MAPE^f^; equivalence testing	Accuracy judged as poor for EE but strong for HR
Bai et al [37]	Healthy adults; aged 18 to 59 years (N=48)	Charge 2	HR; steps not used in the MA (criterion measure=pedometer)	Polar heart rate chest strap	24-hour monitoring in a free-living setting (devices removed during the night)	Correlations; Bland-Altman analyses; MAPE; equivalence testing	Underestimation; accuracy judged as reasonable
Benedetto et al [38]	Healthy adults; aged 25 to 36 years (N=16; observations=9000)	Charge 2	HR	ECG (ProComp Infiniti T7500M)	Maximal 10-minute stationary bicycle test	Multilevel Bland-Altman analyses; ICC^g^	Underestimation; accuracy judged as poor
Boudreaux et al [39]	Healthy adults; aged 18 to 35 years (N=50)	Charge 2; Blaze	HR; EE not used in the MA (absolute value cannot be compiled)	ECG (Quinton 4500)	Structured activity protocol, including stationary cycling and resistance exercises (total time not provided)	MAPE; ICC; Bland-Altman analyses	Underestimation of HR judged as valid depending on the intensities and activities; accuracy of EE judged as inaccurate
Bunn et al [40]	Healthy adults; mean age 26 years (N=20)	Surge	Steps	Video recorded	10-minute walking and running bouts on a treadmill	MAPE; correlations; equivalence testing	Underestimation of steps above standards (MAPE<10%) for the walking bout and overestimation for the running bout; accuracy judged as poor for both intensities
Burton et al [41]	Healthy older adults; age >65 years (N=31)	Charge HR	Steps	Video recorded	2-minute walking tests; 2-week of measures in a free-living environment not used in the MA (criterion measure=accelerometer)	ICC; Bland-Altman analyses	Underestimation of steps; accuracy judged as good
Cadmus-Bertram et al [42]	Healthy adults; aged 30 to 65 years (N=40)	Surge	HR	ECG (type not specified)	10-minute treadmill exercise at 65% of the maximum HR	Multilevel Bland-Altman analyses	Accurate agreement at rest; poor agreement when participant exercised at 65% of their maximum HR; overall accuracy judged as insufficient
Chow et al^h^ [43]	Healthy adults; mean age 24 years (N=31)	Charge HR	Steps	Manual hand counter	3-minute treadmill exercise at varying speeds	ANOVA^i^	Underestimation of steps at slowest speeds; accuracy improved at faster speeds; no clear conclusion about the overall accuracy of the device
Chowdhury et al [44]	Healthy adults; aged 18 to 50 years (N=30)	Charge HR	EE	Indirect calorimetry (COSMED K4b2)	Simulated activities of daily living and structured exercise in laboratory conditions (64-minute in total); 24-hour period in free-living conditions not used in the MA (criterion measure=accelerometers and armband device)	Bland-Altman analyses; mean signed error tests; MAE^j^ tests; correlations; ANOVA; equivalence testing	Underestimation of EE in the 2 conditions; not as consistent as research-grade devices
Claes et al [45]	Healthy adults; aged 18 to 40 years (N=18)	Charge HR	EE; steps	Indirect calorimetry (Jaeger Oxycon Mobile); video recorded	50-minute protocol on a treadmill at various intensities	Paired sample *t* tests; Wilcoxon signed ranks tests; Bland-Altman analyses	Estimation of the 2 outcomes judged as accurate
Herkert et al [46]	Patients with cardiac conditions (N=19)	Charge 2	EE	Indirect calorimetry (Jaeger Oxycon Mobile)	Low- to moderate-intensity walking and cycling activities (protocol duration not provided)	Bland-Altman analyses; ICC	Accuracy judged as poor
Düking et al [47]	Healthy adults; mean age 26 years (N=25)	Versa	HR; EE	Polar HR chest strap; indirect calorimetry (Metamax 3B, CORTEX Biophysik GmbH)	5 minutes of sitting, walking, and running at different velocities and intermittent sprints during 3 minutes performed on a treadmill	Standardized mean bias; standardized typical error of the estimate; coefficient of variation; Pearson correlation	HR should be interpreted with caution because of the high error rate, and the Fitbit should not be used to monitor EE
Dooley et al [15]	Students; aged 18 to 38 years (N=62)	Charge HR	HR; EE	Polar HR chest strap; indirect calorimetry (Parvo Medics TrueOne 2400)	40-minute treadmill protocol performed at various intensities	ANOVA; Bland-Altman analyses; MAPE	Overestimation of HR during light-intensity activities and overestimation of EE during light and moderate intensities; accuracy judged as reasonably accurate to estimate HR but not accurate for EE
Etiwy et al [48]	Patients with cardiac conditions; mean age 62 years (N=80)	Blaze	HR	ECG (type not specified)	15-minute treadmill protocol performed at various intensities	MAPE; Bland-Altman analyses; correlations; mixed model analyses of variance	Underestimation of HR; accuracy judged as probably insufficient among patients with cardiac conditions
Falgoust et al^h^ [49]	Healthy adults; aged 23 to 54 years (N=30)	Charge HR; Surge	Steps	Manual hand counter	2×2 laps on a track at a self-selected walking speed	ANOVA; correlations	Underestimation of steps, more pronounced for the Fitbit Surge than the Charge HR; accuracy judged as insufficient for research purpose
Fokkema et al [50]	Healthy adults; mean age 32 years (N=31)	Charge HR	Steps	Manual hand counter	Two 30-minute treadmill walking bouts at 3 different walking speeds	ICC; MAPE; paired sample *t* tests; Wilcoxon signed-rank tests	Accuracy decreased as walking speed increased; accuracy was judged as not valid for high walking speeds but acceptable for lower walking speeds
Gaynor et al [51]	Patients with respiratory conditions; mean age 34 years (N=15)	Charge HR	HR	ECG (type not specified)	One 15-minute session of continuous cycling on an ergometer and one 15-minute session of interval cycling	Bland-Altman analyses	Underestimation, particularly pronounced during continuous exercise compared with interval training; authors recommended to not use a Fitbit Charge HR for assessing HR during exercise in adults with cystic fibrosis
Gillinov et al [52]	Healthy adults; mean age 38 years (N=50; observations=3985)	Blaze	HR	ECG (type not specified)	24-minute structured exercise protocols on a treadmill, ergometer, and elliptical trainer	Correlations; MAPE; Bland-Altman analyses; mixed model analyses of variance	Accuracy varies with the activities but, overall, judged mostly inaccurate
Gorny et al [16]	Healthy adults; mean age 25 years (N=10; observations=2769)	Charge HR	HR	Polar HR chest strap	3 to 6 hours of normal daily living activities	ICC; Multilevel Bland-Altman analyses	Underestimation, particularly pronounced for higher intensity activities; accuracy inconclusive
Jagim et al [53]	Healthy adults; mean age 24 years (N=20)	Versa	HR; EE	ECG (12-lead CareCenter MD ECG); indirect calorimetry (TrueMax 2400 Metabolic Measurement System, Parvo- Medics)	12-minute graded exercise protocol at speeds of 4.8 km/hour, 7.2 km/hour, 9.6 km/hour, and 12.1 km/hour on a motorized treadmill	Pearson correlation; ANOVAs; MAPE; constant error; Bland-Altman analyses	Underestimation of HR and overestimation of EE; no clear conclusion about the overall accuracy of the device
Jo et al [54]	Healthy adults; mean age 24 years (N=24; observations=87,340)	Charge HR	HR	ECG (Cosmed C12x)	77-minute protocol comprising various activities (treadmill, ergometer, and resistance) performed at two intensities (light and moderate to vigorous)	Correlations; multilevel Bland-Altman analyses; MAPE	Underestimation at the higher ends of the mean HR spectrum; failed to satisfy validity criteria
Lai et al [55]	Patients with Parkinson disease; mean age 64 years (N=31)	Charge 2	Steps	Manual hand counter	6-minute bouts of overground and treadmill walking at a comfortable speed	ICCs; Bland-Altman analyses; MPE^k^	Accurate and precise for overground walking only
Lamont et al^h^ [56]	Patients with Parkinson disease; mean age 69 years (N=33)	Charge HR	HR; steps not used in the MA (criterion measure=accelerometer)	Polar HR chest strap	Six 2-minute walking bouts at various intensities on an indoor track	MAPE; Bland-Altman analyses; paired sample *t* tests	Weakly associated with increases in HR; no clear conclusion about the overall accuracy of the device
Lee et al [57]	Students; mean age 27 years (N=10)	Charge HR	HR	Polar HR chest strap	8-hour continuous monitoring during normal daily activities	Correlations; MAPE; Multilevel analyses of variance	Measurement judged as inaccurate
Modave et al^h^ [58]	Healthy adults in three age groups: 18 to 39 years, 40 to 64 years, 65 to 84 years (N=60)	Surge	Steps	Manual hand counter	Two separate 1000-step walks on a treadmill at a self-selected speed	Multilevel analyses of variance	Underestimation of steps across all age groups
Montes et al [59]	Healthy adults; mean age 25 years (N=40)	Surge	Steps	Manual hand counter	5-minute walking and running free motion and treadmill	MAPE; Bland-Altman analyses; Pearson correlation; ICC	Underestimation of steps for all activities, with walking activities being higher than the running; valid for all conditions except treadmill walking
Montoye et al [60]	Healthy adults; mean age 24 years (N=32)	Charge HR	HR; EE; steps not used in the MA (criterion measure=pedometer)	Pulse oximeter (Nonin PureSAT); indirect calorimetry (Parvo TrueOne 2400)	90-minute structured protocol performed at various intensities in laboratory condition and on a 200 m indoor track	ANOVA; paired sample *t* tests; MAPEs; Bland-Altman analyses	Underestimation of HRs for higher intensity activities and poor estimation of EE
Morris et al [61]	Healthy adults; mean age 29 years (N=47)	Charge HR	EE	Indirect calorimetry (Cosmed K4b2)	15-minute high-intensity workout	ICC; ANOVA; MAPE	Significant underestimation of EE; judged as inaccurate
Muggeridge et al [62]	Healthy adults; mean age 40 years (N=20; *k*=35,639)	Charge 3	HR	Polar HR chest strap	Visit 1: 15-minute sedentary activities, 10-minute cycling on a bicycle ergometer, and incremental exercise test to exhaustion on a motorized treadmill; visit 2: four 15-second maximal sprints on a cycle ergometer and four 30 m to 50 m sprints on a treadmill	Multilevel Bland-Altman analyses; MAPE; Pearson correlation	Accuracy was generally poor, notably, during cycling exercises; underestimation of HR
Nelson and Allen [63]	Healthy adults; mean age 29 years (N=1; *k*=102,740)	Charge 2	HR	ECG (Vrije Universiteit Ambulatory Monitoring System)	24 hours of daily living monitoring	Multilevel Bland-Altman analyses; MAPE; CCC^l^	Slight underestimation; judged as acceptable
Nuss et al [64]	Healthy adults; mean age 24 years (N=20)	Charge 2	EE	Indirect calorimetry (Parvo Medics TrueOne 2400)	Bruce treadmill protocol (maximal)	CCC; MAPE; paired sample *t* tests	Significant underestimation judged as inaccurate
Pasadyn et al^h^ [65]	Healthy adults; mean age 29 years (N=50)	Ionic	HR	ECG (Quinton Q-tel RMS telemetry system)	12-minute treadmill protocol performed at various intensities	CCC; Bland-Altman analyses; mixed model ANOVA	Moderate to high level of accuracy
Powierza et al [66]	Healthy adults; age range 18 to 26 years (N=22)	Charge HR	HR	ECG (MP150, BioPac Systems)	Buffalo Concussion Treadmill Test (maximal)	ICC; multilevel Bland-Altman analyses; MAPE	Small underestimation judged as not accurate for monitoring HR within a narrow range
Pribyslavska et al^h^ [67]	Healthy adults; mean age 26 years (N=34)	Surge	EE	Indirect calorimetry (Oxycon Mobile)	Two 2-minute bouts on an ergometer and treadmill at different intensities	PE^m^; MAPE	Underestimation; accuracy judged as reasonable
Reddy et al [68]	Healthy adults; mean age 28 years (N=20)	Charge 2	HR; EE	Polar HR chest strap; indirect calorimetry (Cosmed K4b2 or Cosmed K5)	Maximal oxygen uptake test, resistance exercises, interval training (27 minutes), and free-living activities (28 minutes)	MAPE; Bland-Altman analyses; correlations	Underestimation; accuracy judged as reasonable
Salazar et al [69]	Healthy adults; mean age 22 years (N=35)	Charge 2	HR	Polar HR strap	12-minute treadmill protocol at different intensities	ANOVA; correlations	Underestimation; accuracy judged as adequate
Shcherbina et al^h^ [70]	Healthy adults; mean age 38 years (N=60)	Surge	HR; EE	ECG (type not specified); indirect calorimetry (Quark CPET, COSMED)	38-minute treadmill and ergometer protocol performed at various intensities	PE; ANOVA; principal component analysis; correlations; Bland-Altman analyses	Measure of HR judged adequate but poor for EE
Siddall et al [71]	Military officer trainees; mean age 23 years (N=20)	Surge	EE	Doubly labeled water	10 days of military training	Correlations; Bland-Altman analyses	Underestimation judged as insufficiently accurate
Sjöberg et al [72]	Adults with chronic pain; mean age 44 years (N=41)	Versa	EE; HR	Indirect calorimetry (Jaeger Oxycon Pro); Polar HR strap	Treadmill walking at three speeds (3.0 km/hour, 4.5 km/hour, and 6.0 km/hour) in the laboratory setting	ICC; ANOVA; Bland-Altman; MAPE	Overestimation of EE; accuracy judged as poor; good agreement for HR that tends to decrease with speed
Stahl et al [73]	Healthy adults; age range 19 to 45 years (N=50; observations=1781)	Charge HR	HR	Polar HR strap	30-minute treadmill protocol performed at different intensities	Correlations; multilevel Bland-Altman analyses; MAPE; ANOVA; equivalence testing	Small underestimation; accuracy judged as adequate
Tam and Cheung [74]	Healthy adults; mean age 32 years (N=30)	Charge HR	Steps	Video recorded	25-minute treadmill protocol performed at different intensities	Paired sample *t* tests; Bland-Altman analyses; correlations; MAPE	Estimation judged as accurate
Tedesco et al [75]	Older adults; mean age 69 years (N=18)	Charge 2	Steps; HR	Video recorded; Polar HR chest strap	3-hour structured protocol involving walking on a treadmill, simulated household, and sedentary activities	Mean bias; MPE; MAPE; MAD^n^; MAE; RMSE^o^; ICC; paired sample *t* tests; Wilcoxon signed-rank test; Bland-Altman analyses	Underestimation of heart rate; deficits in accuracy
Thiebaud et al [76]	Healthy adults; mean age 22 years (N=22)	Surge	HR; EE	ECG (Quinton Q-Stress, version 4.5); indirect calorimetry (Trueone 2400, Parvomedics)	15-minute treadmill protocol performed at various intensities	Correlations; limits of agreement; MAPE; equivalence testing	Underestimation of HR judged as acceptable; overestimation of EE at each speed and judged as insufficiently accurate
Thomson et al [77]	Healthy adults; mean age 24 years (N=30)	Charge HR	HR	ECG (Q-Stress, Mortara)	Bruce treadmill protocol (maximal)	Equivalence testing; CCC; Bland-Altman analyses	Underestimation increasing with intensity; Overall accuracy judged as insufficient
Tophøj et al^h^ [78]	Healthy students; mean age 26 years (N=20)	Surge; Charge HR	Steps	Manual hand counter	800 steps performed on a treadmill	MAPE; ICC; Bland-Altman analyses	Accurate estimation for the Fitbit Surge at higher walking speeds and inaccurate estimations at lower speeds; the Fitbit Charge HR was judged as insufficiently accurate
Wahl et al^h^ [79]	Healthy sport students; mean age 25 years (N=20)	Charge HR	Step count; EE	Manual hand counter; indirect calorimetry (Metamax 3B, CORTEX Biophysik GmbH)	55-minute treadmill protocol at constant and intermittent velocities; outdoor exercise not included in the MA	MAPE; ICC; TE^p^; Bland-Altman analyses	Acceptable level of validity for steps; inaccurate estimation of EE, with overestimation of EE for slower velocities and underestimation of EE for higher velocities
Wallen et al [80]	Healthy participants; mean age 24 years (N=22)	Charge HR	HR; EE; steps	ECG (CASE, GE Healthcare); indirect calorimetry (MetaMax 3B, Cortex); video recorded	58-minute treadmill and ergometer protocol performed at various intensities	Correlations; Bland-Altman analyses	Accurate measure of HR; overestimation of EE, judged as inaccurate; no clear conclusion is proposed for steps
Wang et al^h^ [81]	Healthy participants; mean age 37 years (N=50; observations=1773)	Charge HR	HR	Polar HR chest strap	18-minute treadmill protocol at various intensities	CCC; Wilcoxon signed-rank; Bland-Altman analyses	Adequate estimation of HR at low intensities, suboptimal accuracy during moderate exercise, and underestimated during vigorous exercise; judgment deemed as inaccurate
Xie et al^h^ [82]	Healthy participants; aged 19 to 27 years (N=44)	Surge	Steps; EE; HR not used in the MA (criterion measure=manual estimation)	Indirect calorimetry (Cosmed K4b2); video recorded	Walking, running, and cycling on a 400 m standard track	MAPE; correlations; paired sample *t* tests	High accuracy of measure for steps; inadequate accuracy of EE
Zhang et al [83]	Healthy students; mean age 20 years (N=30)	Charge HR	EE	Indirect calorimetry (TrueOne 2400, Parvo Medics Inc)	6 structured 10-minute exercise bouts on a treadmill at various intensities	Paired sample *t* tests; equivalence testing; correlations; MAPE; Bland-Altman analyses	Estimation of EE judged as adequate during treadmill running

^a^HR: heart rate.

^b^ECG: electrocardiogram.

^c^bpm: beats per minute.

^d^EE: energy expenditure.

^e^MA: meta-analysis.

^f^MAPE: mean absolute percentage error.

^g^ICC: intraclass correlation coefficient.

^h^Studies not included in the meta-analysis.

^i^ANOVA: analysis of variance.

^j^MAE: mean absolute error.

^k^MPE: mean percentage error.

^l^CCC: concordance correlation coefficient.

^m^PE: percentage error.

^n^MAD: median absolute deviation.

^o^RMSE: root mean square error.

^p^TE: typical error.

### Meta-analyses

Table 2 presents the results of the main and sensitivity analyses after removing studies with a high risk of bias (ie, low quality). Regarding heart rate, the pooled estimate of the mean bias between Fitbit devices and criterion measures was −3.39 bpm (*k* comparison=117), indicating an underestimation of the Fitbits compared with criterion measures. The range in population limits of agreement was large, resulting in the 2 methods differing from −24 bpm to 18 bpm across all studies. Underestimation slightly improved when removing low-quality studies (*k* comparison=74) from −3.39 bpm for the main analysis to −2.99 bpm (however, heterogeneity remained similar).

Regarding steps, the mean bias between Fitbit devices and criterion measures was −1.47 steps per minute, indicating an underestimation of the Fitbits compared with the criterion measures (*k* comparison=37). The population limit of agreement was large, ranging from −15 steps per minute to 12 steps per minute across all studies. These differences were more pronounced after removing studies with a low-quality score but with a lower heterogeneity (*k* comparison=19): pooled estimate of −3.11 steps per minute ranged between −13 steps per minute and 7 steps per minute.

**Figure 1:**
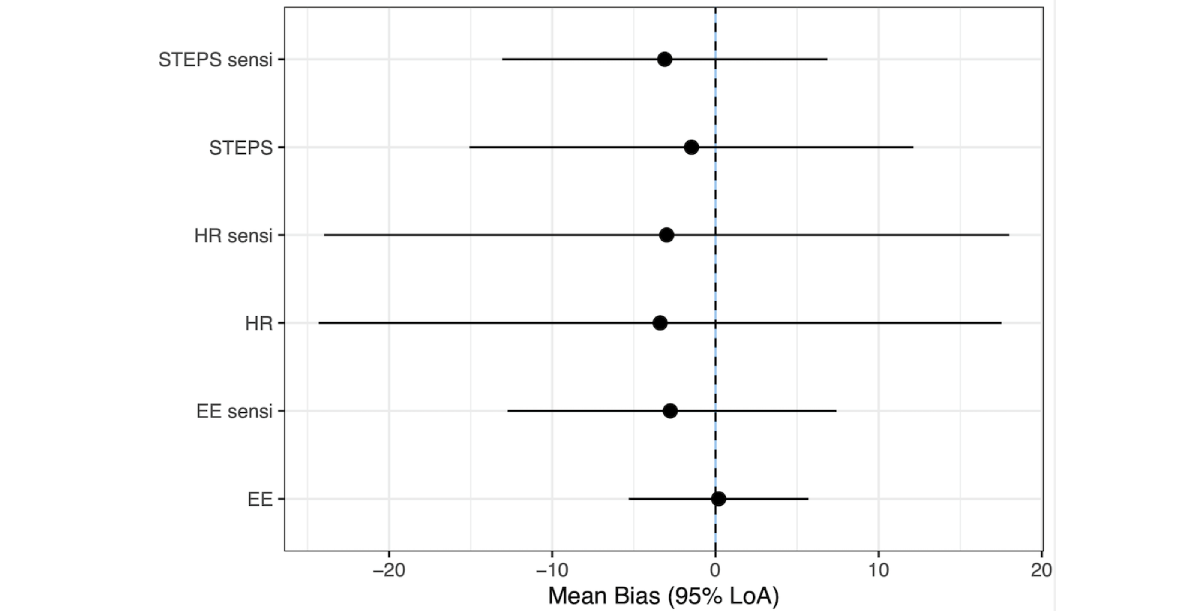
Forest plots for the main and sensitivity analyses. EE: energy expenditure; HR: heart rate; LoA: limits of agreement.

Figure 1 displays the results (main meta-analyses and sensitivity analyses) as a forest plot. Figure 1 highlights the particularly high heterogeneity for heart rate compared with energy expenditure and steps. This heterogeneity is addressed in the following section using a series of subgroup analyses.

**Table 2:** Results of the main meta-analysis.

Analyses		*k* comparisons^a^	Bias^b^, mean (SD^c^)	τ^d^	LoA^e,f^	95% CI^g^
**Main analyses**						
	HR^h^ (bpm^i^)	117	−3.39 (9.91)	11.35	−24.32 to 17.53	−26.36 to 19.58
	EE^j^ (kcal per minute)	49	0.19 (2.53)	0.99	−5.32 to 5.70	−7.23 to 7.61
	Steps (per minute)	37	−1.47 (6.30)	6.50	−15.07 to 12.13	−20.55 to 17.61
**Low-quality studies removed**						
	HR (bpm)	74	−2.99 (9.43)	21.42	−23.99 to 18.01	−27.68 to 21.71
	EE (kcal per minute)	29	−2.77 (4.12)	8.40	−12.75 to 7.41	−15.28 to 9.95
	Steps (per minute)	19	−3.11 (4.32)	6.17	−13.07 to 6.86	−17.27 to 11.06
**Outliers removed**						
	HR (bpm)	116	−3.34 (9.79)	11.35	−24.06 to 17.37	−26.09 to 19.40
	EE (kcal per minute)	48	0.19 (2.38)	0.98	−4.96 to 5.38	−6.68 to 7.06
	Steps (per minute)	36	−1.02 (6.07)	6.17	−14.15 to 12.11	−19.49 to 17.46

^a^*k* comparisons is the number of comparisons between the Fitbits and criterion measures available within studies.

^b^Bias is the pooled estimate of mean differences calculated as Fitbit–criterion measures.

^c^SD is the pooled SD of differences.

^d^τ is the variation in bias between studies.

^e^LoA: limits of agreement.

^f^Lower 95% limit of agreement calculated from pooled estimates of bias and SD of differences with robust variance estimation and upper 95% limit of agreement calculated from pooled estimates of bias and SD of differences with robust variance estimation.

^g^Outer confidence bound for lower 95% limit of agreement and outer confidence bound for the upper 95% limit of agreement.

^h^HR: heart rate.

^i^bpm: beats per minute.

^j^EE: energy expenditure. Regarding energy expenditure, the mean bias between Fitbits and criterion measures was 0.19 kcal per minute, and the range in population limits of agreement was large, between −5 kcal per minute and 6 kcal per minute across participants (*k* comparison=49). This result is somewhat inconsistent with the meta-analysis, excluding low-quality studies (*k* comparison=29), which indicated an underestimation of the Fitbit of −2.77 kcal per minute (population limits of agreement comprise between −13 kcal per minute and 7 kcal per minute).

### Subgroup Analyses

**Figure 2:**
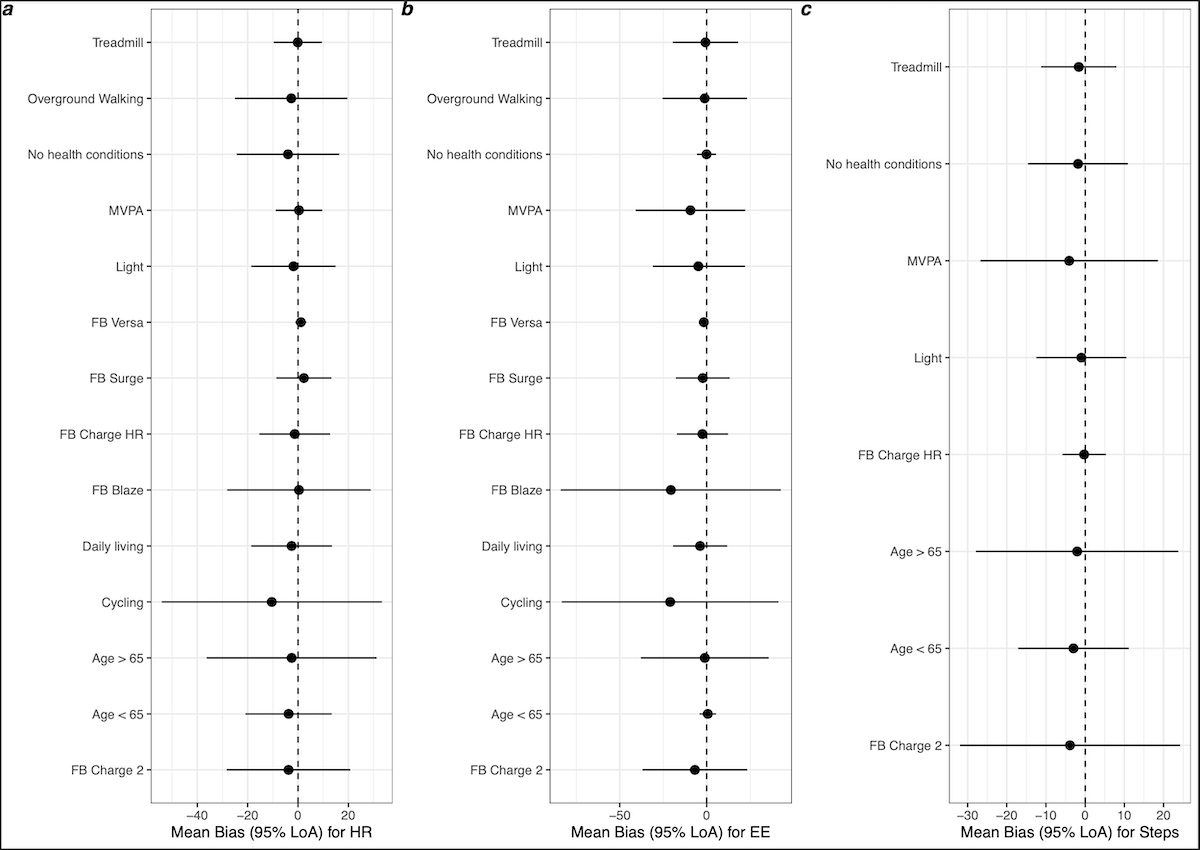
Forest plots for the subgroup analyses. EE: energy expenditure; FB: Fitbit; HR: heart rate; LoA: limits of agreement; MVPA: moderate to vigorous physical activity.

A range of subgroup analyses is presented in Tables S1, S2, and S3 in Multimedia Appendix 4 and can be visualized altogether in Figure 2. Overall, subgroup analyses by population characteristics, intensities, and types of activities, as well as Fitbits’ models, were consistent with the main findings (ie, showing an underestimation of the Fitbits compared with criterion measures in most cases).

Compared with young and middle-aged adults, the results indicated a relatively similar mean bias in the 2 age groups, as well as in the subgroup of participants without health conditions (these results should be considered with caution, given the disproportionately lower number of studies conducted in older adults; *k* comparisons were between 6 and 26). Heterogeneity in these effects (ie, 95% limits of agreement) was systematically lower in younger than in older adults and lower in participants without health conditions, particularly for energy expenditure (Table S1 in Multimedia Appendix 4; Figure 2).

The results of the subgroup meta-analyses for different intensities and types of activities (Table S2 in Multimedia Appendix 4) clearly show a more pronounced underestimation of heart rate and energy expenditure for cycling activities compared with daily living and treadmill activities as well as overground walking. Performance of the device was better (lower heterogeneity) for treadmills than for overground walking. For energy expenditure and steps, the underestimation, and heterogeneity of these effects, were larger for moderate to vigorous intensity activities than for light-intensity activities. Opposite results were observed for heart rate, with more accurate measurements (ie, smaller bias and lower heterogeneity) at moderate to vigorous intensity activities compared with light-intensity activities.

The results of the subgroup meta-analyses by type of device and considering the number of *k* comparisons available by device show that the Fitbit Charge HR presents better performance than other models, notably in comparison with the Fitbit Charge 2 that has been tested in a comparable number of studies (Table S3 in Multimedia Appendix 4). Performance of the Fitbit Charge HR was particularly good for steps, with a mean bias of −0.27 steps per minute ranging between −6 steps per minute and 5 steps per minute. Interestingly, the Fitbit Versa was particularly precise compared with other models (Figure 2); however, this result should be confirmed on the basis of more future validation studies for this specific device.

Figure 2 displays the results of the subgroup meta-analyses for heart rate (Figure 2A), energy expenditure (Figure 2B), and steps (Figure 2C).

## Discussion

### Principal Findings

The results of this systematic review and meta-analysis showed that Fitbit devices are likely to underestimate heart rate, energy expenditure, and steps. This work adds to the current state of evidence for the analytical validity of heart rate, energy expenditure, and steps measured by recent combined-sensing Fitbits compared with criterion measures, many of which are considered *gold standards* or widely used reference standards. This is also the first review to include meta-analyses of Bland-Altman results evaluating the validity of measures of heart rate, energy expenditure, and steps for these devices. Thus, it offers actionable quantitative information to appreciate device validity.

Overall, our systematic review revealed that most authors of the included studies concluded that Fitbits underestimated heart rate, energy expenditure, and steps compared with criterion measures (Table 1). These independent (qualitative) conclusions aligned with the results of our meta-analysis, even in sensitivity and subgroup analyses that considered various aspects of study quality. The fact that results from the authors’ qualitative conclusion (obtained via our systematic review) and this meta-analysis aligned is important, given the heterogeneity of study designs and statistical procedures used in the literature. The underestimation of activity intensity appears consistent with previous systematic reviews, including different brands of activity monitors, older Fitbits, and/or other criterion measures than those considered in this study (see the study by O’Driscoll et al [17] for energy expenditure, the study by Evenson et al [18] for steps, and the studies by Fuller et al [19] and Feehan et al [20] for the 3 outcomes).

However, precisely interpreting the magnitude of this underestimation remains a challenge, as there is little consensus in the literature regarding what constitutes an acceptable magnitude of bias or error. As observed in this systematic review, the interpretations and conclusions from the authors of the included studies were highly variable from one study to another (ie, a result deemed acceptable in one study can be judged as poor in another). Excluding low-quality studies, our pooled estimates indicated that Fitbits underestimate by approximately 3 bpm, 3 steps per minute, and 3 kcal per minute compared with the respective criterion measures. The implications of these differences depend on the nature of the comparisons and on the application. For heart rate, an underestimation of 3 bpm may be an acceptable difference, as the Association for the Advancement of Medical Instrumentation has defined the accuracy of cardiac monitors, heart rate meters, and alarms as a readout error of no greater than +5 and –5 bpm [84]. A similar interpretation can be provided for steps. Assuming that the average 3 steps per minute bias is linear over time and intensities, a 1-hour walk would result in an average underestimation of 180 steps (3 steps × 60 minutes). At a pace of 100 steps per minute (which corresponds to a moderate-intensity walk for the general population [85]), the Fitbit would indicate 5820 steps instead of 6000, which might be judged as a relatively small underestimation of 3% (ie, 5820×100/6000). However, a mean bias of 3 kcal per minute might be met with greater concern. Applying a similar logic as for the steps, after 1 hour of a specific activity, the Fitbit would detect an average of −180 kcal per minute. This is the estimated difference between a 1-hour walk at 3.5 mph to 4.5 mph for a 154 lbs (70 kg) person (respectively 280 kcal per hour and 460 kcal per hour [86]), representing an underestimation of approximately 40% (ie, 280×100/460).

The approximately 3 units of underestimation referred to above may vary largely within participants as well between studies and contexts (as indicated by the large pooled limit of agreement and their CIs, as well as the variation *τ* in bias between studies). According to our subgroup analyses, this heterogeneity is higher (1) in older adults than in younger adults and adults without chronic health conditions, (2) for cycling activities than for other activities, and (3) for the Fitbit Charge 2 than for the Fitbit Charge HR (ie, the 2 devices that received the most attention in the literature). Noticeable results also include reduced heterogeneity (ie, better validity) for energy expenditure in younger adults, heart rate for moderate to vigorous intensities, and Fitbit Charge HR for steps. Other potential differences must be taken with caution, given the number of comparisons (*k*) available per subgroup analysis. Replicating these subgroup analyses with an individual participant meta-analysis approach (ie, meta-analyzing each participant’s estimates instead of the studies’ pooled estimates) would constitute an interesting next step to even more precisely quantify the heterogeneity in these effects. However, this would require a greater number of open-access data sets from researchers in this specific field, which is not the case for now.

This study also highlights the need for ongoing high-quality validation research that uses a greater level of protocol standardization, particularly in regard to the assessment tasks, criterion measures, and reported analyses, following, for instance, the ones recommended in the study by Welk et al [87]. Consensus-building efforts that are focused on methodological rigor among researchers in this field are warranted, as are efforts to establish acceptable ranges of accuracy for the metrics of interest. The adoption of common practices for validation studies would facilitate the conduct of robust meta-analyses with comparable metrics and outcomes. In addition, protocols that systematically isolate a wide range of suggested sources of bias (eg, device movement, arm hair, sweat, skin thickness, skin tone, and adiposity) that may affect the underlying technologies in most wrist-worn multi-sensor devices (ie, accelerometry and photoplethysmography) are needed. Finally, as previously mentioned, the adoption of open science practices, notably data sharing, would greatly facilitate future meta-analyses of individual studies.

### Limitations and Perspectives

This systematic review and meta-analysis is not without limitations. First, we restricted our synthesis to studies of adults, as although the number of studies that include children is growing, there remains a dearth of high-quality studies in this area. Additional research across the age span is needed to close the gap in our understanding of how well the Fitbits measure physical activity in young individuals and older adults. Second, many different statistical strategies and related effect sizes are used to estimate the validity of these devices [37]. Researchers have used, separately or in combination, analysis of variance, correlations (eg, intraclass coefficient correlation), and measures of agreement (eg, Bradley-Blackwood test, Bland-Altman analyses, and mean absolute percentage error). At present, there is no specific framework for meta-analyzing statistics, such as mean absolute percentage error, although it is a preferred metric for understanding validity [37]. Thus, the meta-analysis was restricted to the mean bias and SD from the Bland-Altman analyses. Third, the field of physical activity measurement has yet to establish the magnitude of bias from consumer-level activity monitors that is acceptable or problematic. These classifications are likely contingent on the context in which the devices are used. For example, if one is using a consumer-level activity monitor for self-monitoring within a physical activity promotion intervention, a modest underestimation might not have a large negative impact on the research. However, underestimation within epidemiological surveillance efforts is less than ideal. A consensus regarding the magnitude of error that is either acceptable or unacceptable within a given research context would allow for improved interpretation of the results of validation efforts. Finally, to make comparisons between studies, we retrieved the time spent during each protocol task and converted the absolute number of kcal and steps to kcal per minute and steps per minute. This analytical strategy is not without limitations, notably for energy expenditure. This assumes that energy expenditure is linear over time and over a protocol, which may not be the case.

### Conclusions

Compared with reference standards, recent Fitbit devices are likely to underestimate heart rate, energy expenditure, and steps by an average of three units per minute (ie, steps, bpm, and kcal). Although the expected level of accuracy might vary from one context to another, this underestimation can be acceptable, on average, for steps and heart rate. However, the measurement of energy expenditure may be too inaccurate for some research purposes. The estimation of these measurements varied slightly by the quality of the study, age of the participants, type of activities, and model of Fitbit. Overall, devices were more accurate in younger adults, for treadmills activities (notably, compared with cycling), and for the Fitbit Charge HR (notably, for steps).

## Appendix

Multimedia Appendix 1 PRISMA-P (Preferred Reporting Items for Systematic Review and Meta-Analyses Protocols) checklist.

Multimedia Appendix 2 Search terms.

Multimedia Appendix 3 Flow diagram.

Multimedia Appendix 4 Supplementary tables.

## Abbreviations

bpm: beats per minute
COSMIN: Consensus-Based Standards for the Selection of Health Measurement Instruments
PRISMA-P: Preferred Reporting Items for Systematic Review and Meta-Analyses Protocols
PROSPERO: International Prospective Register of Systematic Reviews
RVE: robust variance estimation
