# Supplementary material for: Accuracy and Precision of Energy Expenditure, Heart Rate, and Steps Measured by Combined-Sensing Fitbits Against Reference Measures: Systematic Review and Meta-analysis
Source: JMIR Mhealth Uhealth. 2022 Apr 13;10(4):e35626. doi: 10.2196/35626 (PMC9047731; doi:10.2196/35626)
Supplement: Multimedia Appendix 2 [file mhealth_v10i4e35626_app2.docx]

**Algorithms**

PubMed:

[((Fitbit) AND (((valid*) OR (accura*) OR (compar*) OR (equival*) OR (agreement*)))) AND (((heart rate) OR (step*) OR (energy expenditure)))]

Embase:

#1 'fitbit'/exp OR 'fitbit'

#2 'validation'/exp OR 'validation' OR 'accuracy'/exp OR 'accuracy' OR 'comparison'/exp OR 'comparison' OR 'equivalent' OR 'agreement'/exp OR 'agreement'

#3 'heart rate'/exp OR 'heart rate' OR 'steps' OR 'energy expenditure'/exp OR 'energy expenditure'

#1 AND #2 AND #3
