# Supplementary material for: Accuracy and Precision of Energy Expenditure, Heart Rate, and Steps Measured by Combined-Sensing Fitbits Against Reference Measures: Systematic Review and Meta-analysis
Source: JMIR Mhealth Uhealth. 2022 Apr 13;10(4):e35626. doi: 10.2196/35626 (PMC9047731; doi:10.2196/35626)
Supplement: Multimedia Appendix 3 [file mhealth_v10i4e35626_app3.docx]

**Supplemental material #3**. Flow diagram
