# Supplementary material for: Accuracy and Precision of Energy Expenditure, Heart Rate, and Steps Measured by Combined-Sensing Fitbits Against Reference Measures: Systematic Review and Meta-analysis
Source: JMIR Mhealth Uhealth. 2022 Apr 13;10(4):e35626. doi: 10.2196/35626 (PMC9047731; doi:10.2196/35626)
Supplement: Multimedia Appendix 4 [file mhealth_v10i4e35626_app4.docx]

**Table S1.** Results of the sub-group meta-analyses for participants’ characteristics

|  | *k* comparisons | Mean  Bias | Mean SD | τ | LoA_L_ | LoA_U_ | CI_L_ | CI_U_ |
| --- | --- | --- | --- | --- | --- | --- | --- | --- |
| Age < 65 yo | | | | | | | | |
| HR (bpm) | 90 | -3.73 | 7.57 | 16.18 | -20.87 | 13.41 | -23.56 | 16.10 |
| EE (kcal/min) | 37 | 0.60 | 2.16 | 1.06 | -4.19 | 5.38 | -6.02 | 7.21 |
| Steps (/min) | 36 | -2.99 | 6.17 | 11.69 | -17.10 | 11.13 | -19.99 | 14.02 |
| Age > 65 yo | | | | | | | | |
| HR (bpm) | 28 | -2.51 | 14.76 | 66.83 | -36.26 | 31.24 | -45.62 | 40.61 |
| EE (kcal/min) | 6 | -1.12 | 18.00 | 14.15 | -37.89 | 35.66 | -82.65 | 80.42 |
| Steps (/min) | 6 | -2.05 | 12.11 | 20.28 | -27.88 | 23.79 | -45.16 | 41.07 |
| No health conditions | | | | | | | | |
| HR (bpm) | 107 | -3.98 | 9.69 | 10.24 | -24.38 | 16.42 | -26.34 | 18.38 |
| EE (kcal/min) | 43 | -0.12 | 2.51 | 1.30 | -5.63 | 5.39 | -7.50 | 7.26 |
| Steps (/min) | 35 | -1.82 | 5.85 | 6.45 | -14.59 | 10.93 | -19.75 | 16.10 |

**Note.** HR = heart rate; EE = energy expenditure; k comparisons = number of comparisons between the Fitbits and criterion measures available within-studies. Mean Bias = pooled estimate of mean difference calculated as ‘Fitbit - criterion measures’; Mean SD = pooled standard deviation of differences; τ = variation in bias between studies; LoA_L_ = lower 95% limit of agreement calculated from pooled estimates of bias and SD of differences with robust variance estimation; LoA_u_ = upper 95% limit of agreement calculated from pooled estimates of bias and SD of differences with robust variance estimation; CI_L_ = outer confidence bound for lower 95% limit of agreement; CI_U_ = outer confidence bound for upper 95% limit of agreement; NA = meta-analyses not performed because ‘k comparisons’ < 4.

**Table S2.** Results of the sub-group meta-analyses for intensities and type of activities

|  | *k* comparisons | Mean  Bias | Mean SD | τ | LoA_L_ | LoA_U_ | CI_L_ | CI_U_ |
| --- | --- | --- | --- | --- | --- | --- | --- | --- |
| Light intensity |  |  |  |  |  |  |  |  |
| HR (bpm) | 42 | -1.80 | 5.73 | 37.15 | -18.56 | 14.96 | -23.21 | 19.60 |
| EE (kcal/min) | 25 | -4.83 | 12.67 | 15.46 | -31.02 | 22.05 | -40.95 | 31.99 |
| Steps (/min) | 8 | -0.97 | 5.26 | 5.51 | -12.48 | 10.55 | -24.52 | -25.56 |
| Moderate to vigorous intensities | | | | | | | | |
| HR (bpm) | 41 | 0.39 | 4.47 | 1.54 | -8.83 | 9.66 | -12.76 | 13.61 |
| EE (kcal/min) | 25 | -9.36 | 14.66 | 34.46 | -40.95 | 22.22 | -46.66 | 27.94 |
| Steps (/min) | 7 | -4.08 | 10.15 | 25.27 | -26.74 | 18.57 | -36.07 | 27.90 |
| Cycling |  |  |  |  |  |  |  |  |
| HR (bpm) | 18 | -10.42 | 20.25 | 67.63 | -54.13 | 33.29 | -65.57 | 44.72 |
| EE (kcal/min) | 4 | -21.03 | 30.26 | 54.35 | -83.32 | 41.26 | -112.86 | 70.80 |
| Daily living |  |  |  |  |  |  |  |  |
| HR (bpm) | 17 | -2.54 | 7.30 | 10.85 | -18.56 | 13.48 | -24.27 | 19.19 |
| EE (kcal/min) | 4 | -3.80 | 6.64 | 16.62 | -19.39 | 11.79 | -38.93 | 31.34 |
| Steps (/min) | <4 | NA | | | | | | |
| Treadmill |  |  |  |  |  |  |  |  |
| HR (bpm) | 55 | -0.10 | 4.42 | 3.46 | -9.69 | 9.50 | -11.89 | 11.69 |
| EE (kcal/min) | 26 | -0.72 | 8.30 | 19.19 | -19.50 | 18.05 | -23.62 | 22.17 |
| Steps (/min) | 13 | -1.63 | 4.22 | 5.31 | -11.25 | 7.99 | -14.40 | 11.14 |
| Overground walking |  |  |  |  |  |  |  |  |
| HR (bpm) | 8 | -2.67 | 10.80 | 7.96 | -25.01 | 19.67 | -31.07 | 25.75 |
| EE (kcal/min) | 4 | -1.16 | 11.40 | 17.70 | -25.46 | 23.14 | -54.94 | 52.63 |
| Steps (/min) | <4 | NA | | | | | | |

**Note.** HR = heart rate; EE = energy expenditure; k comparisons = number of comparisons between the Fitbits and criterion measures available within-studies. Mean Bias = pooled estimate of mean difference calculated as ‘Fitbit - criterion measures’; Mean SD = pooled standard deviation of differences; τ = variation in bias between studies; LoA_L_ = lower 95% limit of agreement calculated from pooled estimates of bias and SD of differences with robust variance estimation; LoA_u_ = upper 95% limit of agreement calculated from pooled estimates of bias and SD of differences with robust variance estimation; CI_L_ = outer confidence bound for lower 95% limit of agreement; CI_U_ = outer confidence bound for upper 95% limit of agreement; NA = meta-analyses not performed because ‘k comparisons’ < 4.

**Table S3.** Results of the sub-group meta-analyses for the Fitbits’ models

|  | *k* comparisons | Mean  Bias | Mean SD | | τ | LoA_L_ | LoA_U_ | | CI_L_ | CI_U_ |
| --- | --- | --- | --- | --- | --- | --- | --- | --- | --- | --- |
| Fitbit Charge HR |  |  |  | |  |  |  | |  |  |
| HR (bpm) | 42 | -1.30 | 5.25 | | 21.77 | -15.35 | 12.74 | | -19.07 | 16.46 |
| EE (kcal/min) | 25 | -2.47 | 4.90 | | 30.01 | -17.17 | 12.24 | | -24.55 | 19.62 |
| Steps (/min) | 10 | -0.27 | 2.21 | | 2.83 | -5.83 | 5.29 | | -9.20 | 8.66 |
| Fitbit Blaze |  |  |  | |  |  |  | |  |  |
| HR (bpm) | 10 | 0.38 | 13.34 | | 23.03 | -28.12 | 28.87 | | -36.90 | 37.65 |
| EE (kcal/min) | 4 | -20.62 | 30.55 | | 68.15 | -83.92 | 42.68 | | -115.42 | 74.19 |
| Steps (/min) | <4 | NA | | | | | | | | |
| Fitbit Charge 2 |  |  |  | |  |  |  | |  |  |
| HR (bpm) | 39 | -3.76 | 10.95 | | 30.88 | -28.32 | 20.79 | | -32.72 | 25.19 |
| EE (kcal/min) | 23 | -6.82 | 14.22 | | 24.88 | -36.97 | 23.32 | | -43.60 | 29.95 |
| Steps (/min) | 7 | -3.87 | 13.60 | | 12.02 | -31.95 | 24.21 | | -57.25 | 49.51 |
| Fitbit Surge |  |  |  | |  |  |  | |  |  |
| HR (bpm) | 13 | 2.32 | 5.00 | | 4.82 | -8.61 | 13.25 | | -14.73 | 19.36 |
| EE (kcal/min) | 4 | -2.28 | 6.94 | | 12.01 | -17.79 | 13.23 | | -37.80 | 33.24 |
| Steps (/min) | <4 | NA | | | | | | | | |
| Fitbit Versa |  |  | | | | | | | | |
| HR (bpm) | 10 | 1.13 | 0.83 | 0.52 | | -1.06 | | 3.32 | -2.91 | 5.17 |
| EE (kcal/min) | 4 | -1.66 | 0.44 | 0.33 | | -3.10 | | -0.21 | -5.17 | 1.86 |
| Steps (/min) | <4 | NA | | | | | | | | |

**Note.** HR = heart rate; EE = energy expenditure; k comparisons = number of comparisons between the Fitbits and criterion measures available within-studies. Mean Bias = pooled estimate of mean difference calculated as ‘Fitbit - criterion measures’; Mean SD = pooled standard deviation of differences; τ = variation in bias between studies; LoA_L_ = lower 95% limit of agreement calculated from pooled estimates of bias and SD of differences with robust variance estimation; LoA_u_ = upper 95% limit of agreement calculated from pooled estimates of bias and SD of differences with robust variance estimation; CI_L_ = outer confidence bound for lower 95% limit of agreement; CI_U_ = outer confidence bound for upper 95% limit of agreement; NA = meta-analyses not performed because ‘k comparisons’ < 4.
